# Cost-effectiveness of linezolid to ventilator-associated pneumonia in Colombia

**DOI:** 10.1186/s12879-023-08961-y

**Published:** 2024-01-18

**Authors:** Jefferson Antonio Buendía, Diana Guerrero Patiño, Andrés Felipe Zuluaga Salazar

**Affiliations:** 1 Department of Pharmacology and Toxicology, Facultad de Medicina, Universidad de Antioquia, Medellin, Colombia; 2 Research Group in Pharmacology and Toxicology, University of Antioquia, Medellín, Colombia; 3 Laboratorio Integrado de Medicina Especializada (LIME), Facultad de Medicina, IPS Universitaria, Universidad de Antioquia, Antioquia, Colombia

**Keywords:** Cost-effectiveness analysis, Decision analysis

## Abstract

**Introduction:**

Ventilator-associated pneumonia (VAP) is a prominent cause of morbidity and mortality in intensive care unit (ICU) patients. Due to the increase in Methicillin resistant Staphylococcus aureus infection, it is important to consider other more effective and safer alternatives compared to vancomycin. This motivates evaluating whether the use of an apparently more expensive drug such as linezolid can be cost-effective in Colombia.

**Methods:**

A decision tree was used to simulate the results in terms of the cost and proportion of cured patients. In the simulation, patients can receive antibiotic treatment with linezolid (LZD 600 mg IV/12 h) or vancomycin (VCM 15 mg/kg iv/12 h) for 7 days, patients they can experience events adverse (renal failure and thrombocytopenia). The model was analyzed probabilistically, and a value of information analysis was conducted to inform the value of conducting further research to reduce current uncertainties in the evidence base. Cost-effectiveness was evaluated at a willingness-to-pay (WTP) value of US$5180.

**Results:**

The mean incremental cost of LZD versus VCM is US$-517. This suggests that LZD is less costly. The proportion of patients cured when treated with LZD compared with VCM is 53 vs. 43%, respectively. The mean incremental benefit of LZD versus VCM is 10 This position of absolute dominance (LZD has lower costs and higher proportion of clinical cure than no supplementation) is unnecessary to estimate the incremental cost-effectiveness ratio. There is uncertainty with a 0.999 probability that LZD is more cost-effective than VCM. Our base‐case results were robust to variations in all assumptions and parameters.

**Conclusion:**

LNZ is a cost-effective strategy for patients, ≥ 18 years of age, with VAP in Colombia- Our study provides evidence that can be used by decision-makers to improve clinical practice guidelines.

**Supplementary Information:**

The online version contains supplementary material available at 10.1186/s12879-023-08961-y.

## Introduction

Ventilator-associated pneumonia (VAP) is a significant cause of illness and death in patients receiving intensive care in hospitals. The occurrence of VAP varies between 10 and 41.7 cases per 1000 ventilator days, with mortality rates ranging from 16 to 94% in developing countries [1]. Gram-negative bacilli are the most common pathogens (41–92%), followed by Gram-positive cocci (6–58%) [1]; which aligns with findings from studies conducted in North America and Europe [2]. The rise in VAP cases is also associated with increased antibiotic usage and the emergence of multidrug-resistant organisms such as Pseudomonas aeruginosa, Acinetobacter baumanii, and Staphylococcus aureus [3]. Methicillin resistant Staphylococcus aureus (MRSA) as a variable etiologic agent of VAP with rates of up to 73% [4]. Early initiation of antibiotic treatment is recommended for VAP patients, including dual coverage against gram-negative bacteria and S. aureus [5]. The standard protocol for managing VAP involves the use of vancomycin (VCM) due to the high risk of MRSA, which is typically covered by most health insurance plans in developing countries [5]. Another effective drug option available is linezolid (LZD), an antibiotic belonging to the oxazolidinone group, which offers better safety and penetration into lung tissue [6].

Several clinical studies have compared vancomycin and linezolid. A recent meta-analysis of seven randomized controlled trials (RCTs) involving 1239 patients and eight retrospective cohort or case–control studies (CSs) involving 6125 patients revealed that patients treated with LZD had significantly higher rates of clinical cure and microbiological eradication (clinical cure: risk ratio (RR) = 0.81, 95% confidential interval (CI) = 0.71–0.92; microbiological eradication: RR = 0.71, 95% CI = 0.62–0.81) [7]. No significant differences in adverse events were observed between VCM and LZD in CSs (thrombocytopenia: odds ratio (OR) = 0.95, 95% CI = 0.50–1.82; nephrotoxicity: OR = 1.72, 95% CI = 0.85–3.45) [7].

Given the increasing prevalence of MRSA in hospital settings, it is crucial to consider alternative treatments that are more effective and safer than vancomycin. This highlights the need to evaluate whether linezolid, despite being a potentially more expensive drug, can be cost-effective in Colombia. Existing international publications provide cost information; however, they do not incorporate data from recent clinical investigations and cannot be directly applied to the Colombian healthcare system.

Since linezolid is already available on the market and has a well-established track record of effectiveness and safety, providing information on its efficiency will undoubtedly facilitate informed decisions regarding its inclusion in clinical practice guidelines. The value of an economic evaluation in the current context extends beyond determining cost-effectiveness; it also provides insights into other outcomes that are vital for estimating the impact of such an intervention on public health, such as cost savings per patient treated with this drug.

## Materials and methods

### Population objective

Hospitalized patients, ≥ 18 years of age, with VAP. It was assumed that Colombian patients are similar in physical characteristics and response to treatment. the patients of the studies clinical.

### Perspective

The study was carried out from the perspective of the third payer. Only direct medical costs were taken.

### Comparators

We compared the use of linezolid (600 mg IV/12 h) vs. vancomycin (15 mg/kg iv/12 h) for 7 days in the treatment of VAP due to MRSA.

### Horizon temporary

The horizon was 30 days, following the design of clinical studies, where both efficacy and safety events were considered after the end of treatment.

### Rate of discount

Due a that the horizon it is less than one year, it was not applied discount on costs or health outcomes.

### Unit of result

Costs were expressed in 2022 Colombian pesos (1 USD = $ 4,890) and health outcomes as a proportion of patients cured (individuals with resolution of infectious picture without effects secondary attributable to the antibiotic received).

### Structure of model

**Fig. 1:**
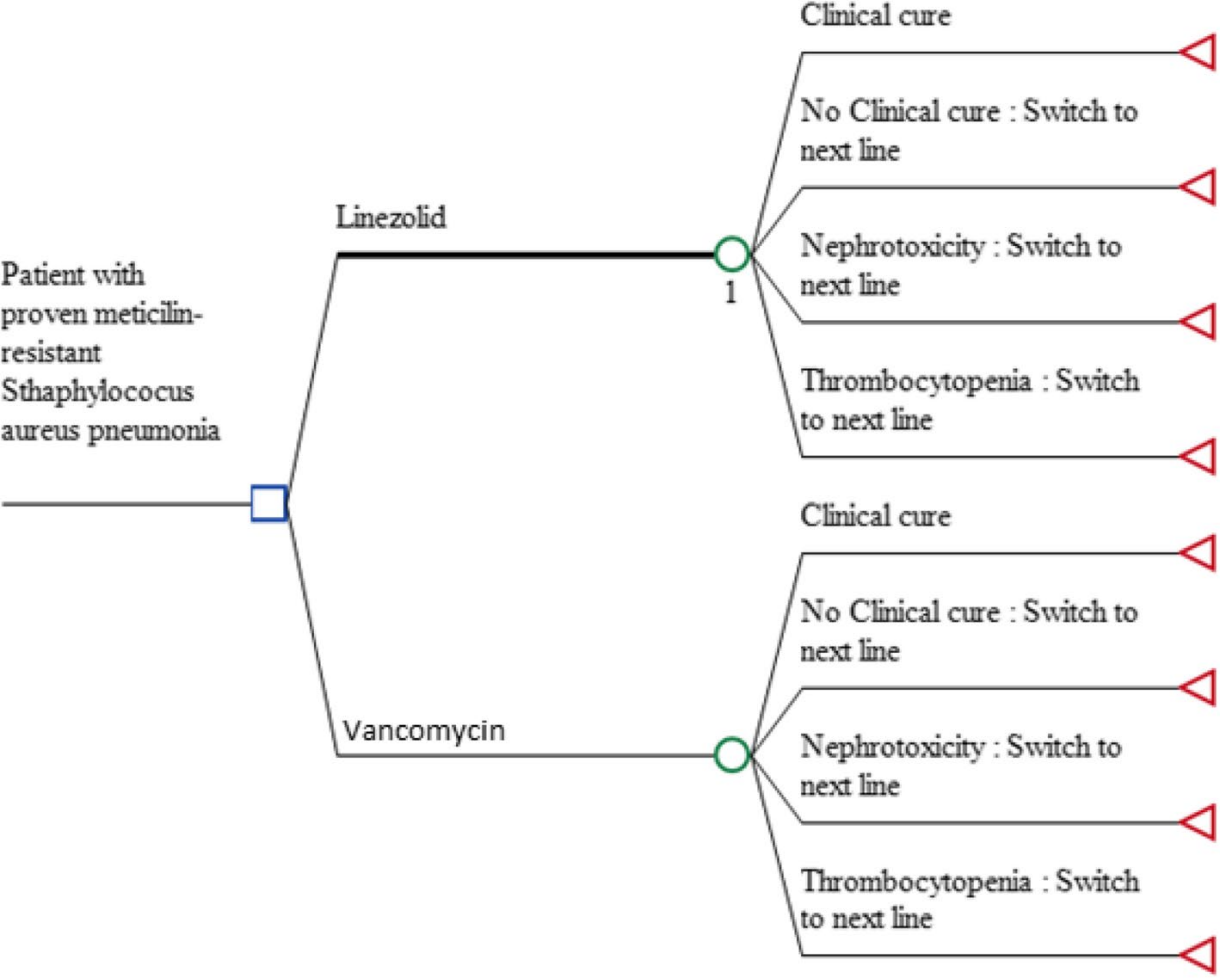
Decision tree model

A decision tree was built (Fig. 1) using TreeAge Pro 2022 (Williamstown, MA: TreeAge Software, Inc) to simulate the results in terms of the proportion of cured patients. This study using published data to build this mathematical model, not human participants were directly involved in the study. In the simulation, patients can receive antibiotic treatment with linezolid (600 mg IV/12 h) or vancomycin (15 mg/kg iv/12 h) for 7 days, patients they can experience events adverse (renal failure and thrombocytopenia). With this decision tree we estimated the proportion of cured patients after completing the treatment. If the treatment fails to either cause an adverse event, the patient will go to a second line of treatment with a duration approximate of 7 days.

### Assumptions of model

The assumptions considered for the building of model are the following:

- Patient Survival: It is assumed that the patient will survive the antibiotic treatment for the specified time horizon of the economic evaluation.
- Exclusion of Death as an Outcome: The outcome of death was not included in the analysis, as clinical evidence does not demonstrate appreciable differences among the various studies [7].
- Impact of Second Line Treatment: For simplicity, it is assumed that the second line of treatment will only affect the expected costs. The primary objective of the economic evaluation is to estimate the proportion of patients cured with the first line of treatment.
- Treatment Switch: It is assumed that if linezolid fails, the patient will receive a second line of treatment with vancomycin, and vice versa.
- Time Horizon and Renal Replacement Therapy: Considering the time horizon of the economic evaluation, it is assumed that patients requiring renal replacement therapy, such as initial hemodiafiltration or acute hemodialysis, will undergo one month of dialysis.

### Data of effectiveness and security

Relative risk (RR) data were obtained from a systematic review and meta-analysis of seven randomized clinical trials (RTCs). The findings indicated a significant increase in both clinical cure and microbiological eradication rates among patients treated with LZD (clinical cure: risk ratio (RR) = 0.81, 95% confidential interval (CI) = 0.71–0.92) [7]. As the RR data were not specific to the Colombian population, a probabilistic sensitivity analysis was conducted as recommended by the Consolidated Health Economic Evaluation Reporting Standards (CHEERS) statement [8]. This sensitivity analysis incorporated the confidence interval of the relative risk mentioned above. Additionally, for the transition probabilities, a variation of ± 25% from the central value defined in the base case was applied [9]. For this economic evaluation was adopted the definition of VAP of IDSA 2016 [10] and clinical cure used in the metanalysis of Kato H, et at (defined as the resolution of pneumonia symptoms and signs of infections such as radiographic findings, excluding patients with pneumonia relapses) [7].

### Cost analysis

The cost data used in this study were obtained from a previously published cost-disease study on VAP conducted in Colombia [11], Table 1. Table 1 of the study provided detailed information on the costs. The study employed a bottom-up methodology, where cost-generating events such as direct health service consumption, diagnostic tests, and drugs were identified based on national clinical guidelines. The quantity and frequency of resource utilization were estimated through the cluster nominal method, using expert opinions. The cost figures were derived from national list prices (ISS and SISMED). In the sensitivity analysis, a range of 25% to 50% was applied to account for potential variations [12]. All costs were adjusted for inflation to account for 2022 Colombian pesos. We used the average exchange rate of 2022 to transform Colombian pesos into U.S. dollars (currency rate: US$1.00 = COP$ 4,000) [13]. The incremental cost-effectiveness ratio (ICER) was calculated using the following formulae:

**Table 1:** Model inputs

Name	Value	Distribution	Paramether 1	Paramether 2	Source
Probability of clinical cure with vancomycin	0.43	Beta	α = 0,46	β = 0,62	[7]
Relative risk of clinical cure of linezolid	1.24	LogNormal	Mean = 0,21	standard deviation = 0,07	[7]
Probability of nephrotoxicity with linezolid	0.08	Beta	α = 13,30	β = 60,59	[9]
Probability of nephrotoxicity with vancomycin	0.18	Beta	α = 14,80	β = 170,20	[9]
Mortality with vancomycin	0.004	Beta	α = 15,94	β = 3969,06	[9]
Mortality with linezolid	0.005	Beta	α = 15,93	β = 3169,08	[9]
Cost f Treatment with linezolid for 7 days	$26	Gamma	α = 1,00	λ = 0,04	[11]
Cost f Treatment with vancomycin for 7 days	$1.28	Gamma	α = 1,64	λ = 1,28	[11]
Cost of stay (ICU and room standard)	$473	Gamma	α = 1,00	λ = 0,0021	[11]
Cost of renal failure	$2461	Gamma	α = 1,00	λ = 0,0004	[11]
Cost of thrombocytopenia	$46	Gamma	α = 1,00	λ = 0,02	[11]
Probability of thrombocytopenia with linezolid	0.16	Beta	α = 13.6	β = 71.4	[9]
Probability of thrombocytopenia with vancomycin	0.13	Beta	α = 14,05	β = 94,03	[9]

$$\frac{\begin{array}{c}Expected\;annual\;cost\;per\;patient\;with\;Linezolid- \\ Expected\;annual\;cost\;per\;patient\;with\;vancomycin \end{array}}{\begin{array}{c}Proportion\;of\;patients\;cured\;with\;Linezolid- \\ Proportion\;of\;patients\;cured\;with\;vancomycin\end{array}}$$

Also, we estimated the net monetary benefit (NMB). NMB represents the value of an intervention in monetary terms [14]. NMB is calculated as (incremental benefit x threshold) – incremental cost. Incremental NMB measures the difference in NMB between alternative interventions, a positive incremental NMB indicating that the intervention is cost-effective compared with the alternative at the given willingness-to-pay threshold. Further analysis was conducted to determine the expected value of perfect information (EVPI) and the perfect parameter information (EVPPI) [15]. The EVPI and EVPPI calculations establish a theoretical upper limit on the value of further research to reduce current uncertainties surrounding all the model input parameters and individual or specific groups of input parameters, respectively. Nonparametric regression-based method for estimating partial EVPI and EVPPI, using the using the Sheffield Accelerated Value of Information (SAVI) tool [16]. The payer strategy-specific burden (PSB) and payer uncertainty burden (PUB) were calculated to reflect the payer's financial risks using the method described by Grimm et al. [17].

### Sensitivity analysis

We conducted a comprehensive sensitivity analysis to assess the robustness of our findings. The results of the one-way sensitivity analysis are presented in a tornado diagram, specifically focusing on the incremental net monetary benefit (NMB). Additionally, a probabilistic sensitivity analysis was performed to account for parameter uncertainties. In this analysis, random sampling was carried out from the distributions of each parameter. The beta distribution was used for relative risk and utilities, while the gamma distribution was employed for costs (see Table 1). To calculate the expected costs and quality-adjusted life years (QALYs) for each treatment strategy, we employed a second-order Monte Carlo simulation with 10,000 replications of each parameter. This simulation allowed us to derive the expected cost-utility for each strategy. To represent decision uncertainty, we plotted the cost-effectiveness and acceptability frontiers. All analyses were conducted using Microsoft Excel.

## Results

### Cost-effectiveness plane

**Fig. 2:**
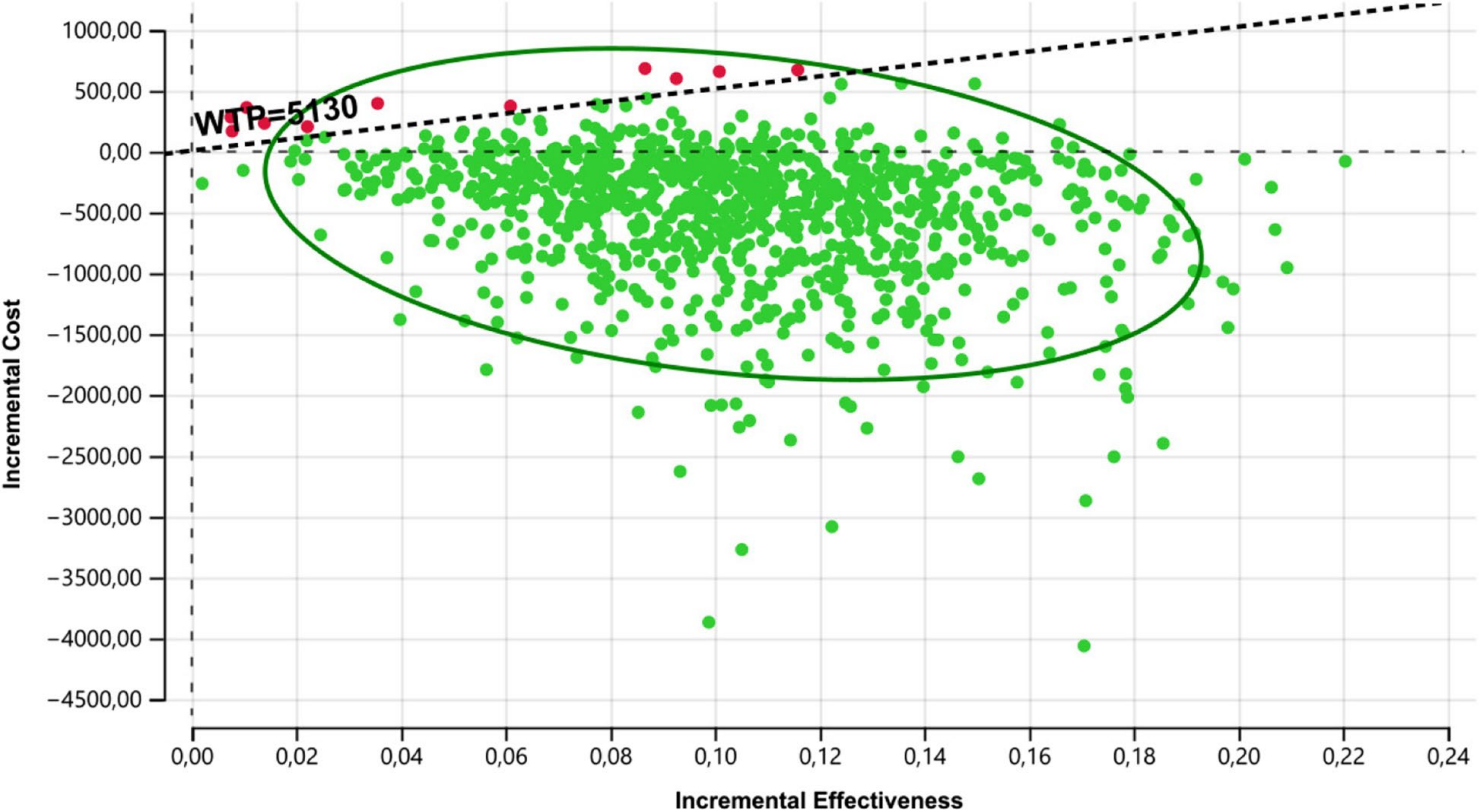
Tornado diagram

The cost-effectiveness plane shows the standardized cost-effectiveness plane per person based on 10,000 model runs in which uncertain model parameters were varied simultaneously in a probabilistic sensitivity analysis. The willingness-to-pay threshold is shown as a degree line, Fig. 2.

The mean incremental cost of LZD versus VCM is US$-517. This suggests that LZD is less costly. The incremental cost is uncertain because the model parameters are uncertain. The 97.5% credible interval for the incremental cost is (US$ -1933.07, £ 289.19). The probability that LZD is cost saving compared to VCV is 0.882.

The proportion of patients cured when treated with LZD compared with VCM is 53 vs. 43%, respectively. The mean incremental benefit of LZD versus VCM is 10%. Again, there is some uncertainty due to model parameters, with the 95% credible interval for the incremental benefit ranging from (0.034 to 0.18). The probability that LZD is more beneficial than VCM is 0.999. This position of absolute dominance (LZD has lower costs and higher proportion of clinical cure than no supplementation) is unnecessary to estimate the incremental cost-effectiveness ratio. There is uncertainty with a 0.999 probability that LZD is more cost-effective than VCM.

**Fig. 3:**
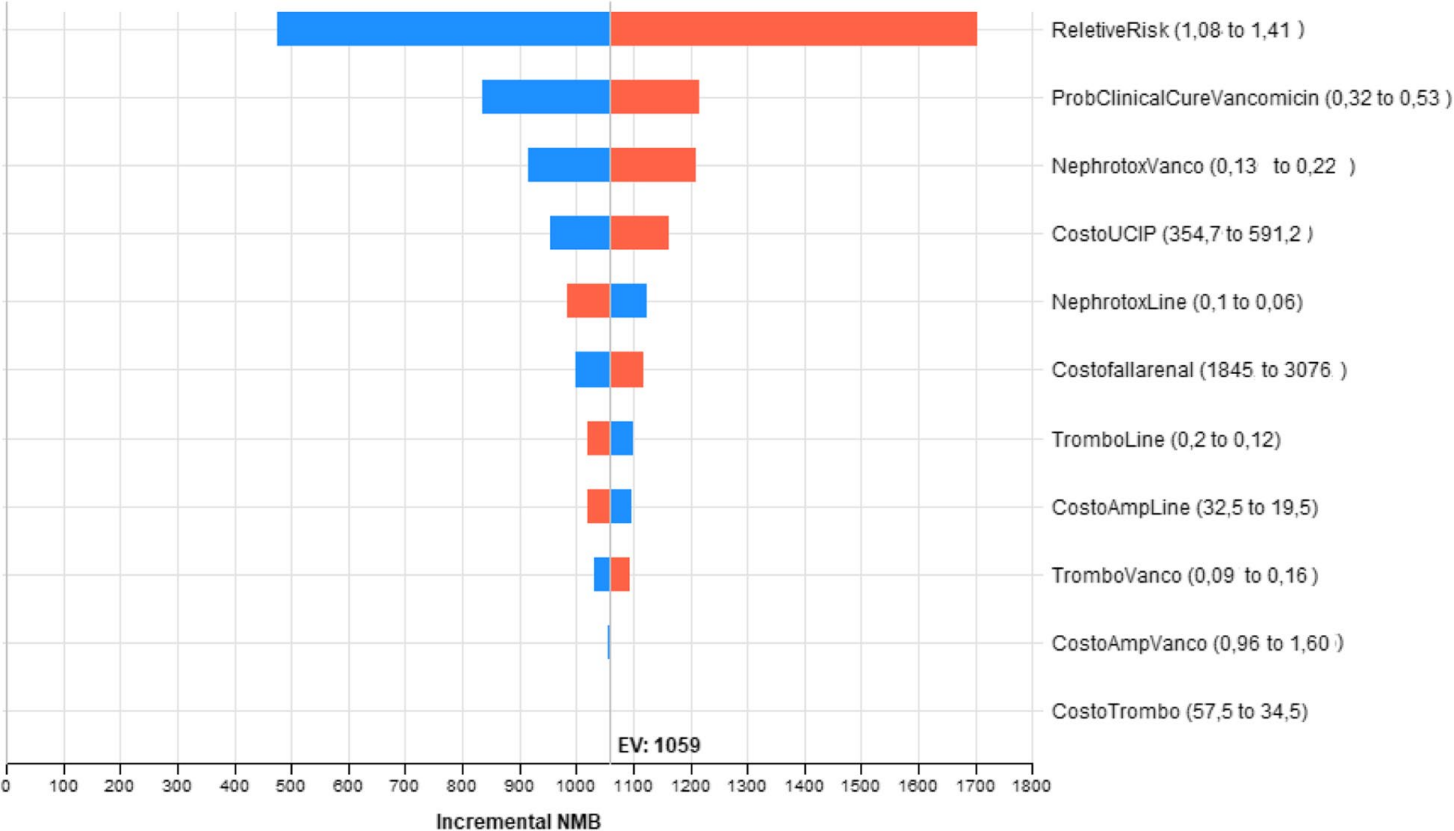
Cost effectiveness plane

In the deterministic sensitivity analyses, our base case results were robust to variations in utilities, transition probabilities, relative risk, and cost; see Fig. 3. LZD was determined to be a cost-effective strategy, since the net monetary benefit was always positive over all the ranges evaluated of each parameter.

### Cost-effectiveness acceptability curve

**Fig. 4:**
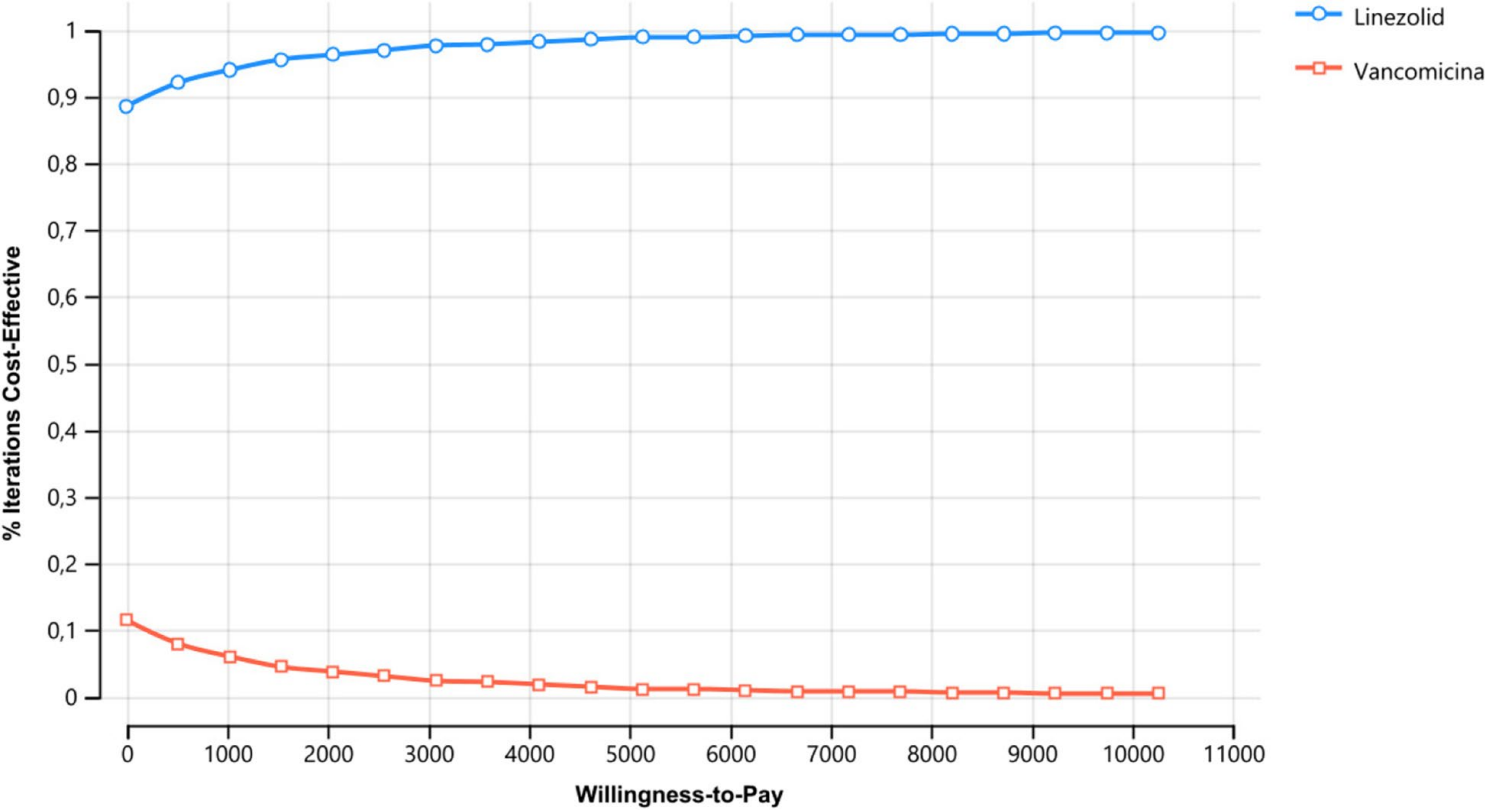
Cost-effectiveness acceptability curve

The Cost-Effectiveness Acceptability Curve (CEAC) shows the probability that all strategies are cost-effective at varying thresholds. The results show that at a threshold value for cost-effectiveness of US$ 5130 per patient cured, the strategy with the highest probability of being most cost-effective is LZD, with a probability of 0.99, Fig. 4.

### Net benefit

Net benefit is a calculation that puts discounted lifetime costs (US$) and discounted lifetime QALYs onto the same scale. The strategy with highest expected net benefit is LZD supplementation with a mean incremental net monetary benefit of USD $1048, with a 95% credible interval of USD $1009 to USD $1088. The distribution of net monetary benefit can be seen in the Supplementary material.

### Expected value of perfect information

The overall EVPI per person affected by the decision is estimated to be USD $2.22, and if the number of patients with VAP per year is 1000, then the overall EVPI per year is USD $225 for Colombia and $182 for VAP due to MRSA in Colombia [4]. If the decision relevance horizon is 10 years, then the overall expected value of removing decision uncertainty for Colombia would be USD $22 253. At WTP of USD $5,180 is not efficient to conduct more research about parameters of the model validated the confidence in our results, Supplementary material. Partial EVPI enables identification of those parameters that particularly contribute to decision uncertainty. The EVPPI was highest (USD $0.07 per person) for the LNZ; the EVPPI for other parameters were USD $0 at WTP of USD $5,180.

### Payer strategy-specific and uncertainty burdens

The payer strategy-specific burden (PSB) reflects the payer's financial risks. The PSB indicates to the payer the risk of choosing an option that is not the most cost-effective option. The value of PBS for VMZ was USD $1036.The population PSB for 1000 annual cases for VAP in Colombia was US$1040 for VMZ, while LNZ, the most cost-effective strategy, reduced the PSB to zero and eliminated any decision uncertainty.

## Discussion

In Colombia, for patients aged 18 and above with ventilator-associated pneumonia (VAP), LNZ has been identified as a cost-effective treatment strategy. Not only does LNZ offer lower costs, but it also provides greater clinical benefits, leading to a higher proportion of cured patients compared to VCM. Our mathematical simulation, based on a cost-effectiveness threshold of USD $5,180 per quality-adjusted life year (QALY), indicated that LNZ had the highest probability (0.99) of being the most cost-effective strategy. These findings hold significance for healthcare service planning, updating clinical practice guidelines, and incorporating new antibiotics into the benefit plans of health insurers in developing countries facing escalating issues of bacterial resistance due to nosocomial infections, such as the case in Colombia [4, 18].

Despite the higher cost of LNZ compared to VMC, our econometric model takes into account the positive effects associated with clinical cure, thereby mitigating the impact of this increased marginal cost. Our findings align with previous economic evidence. Mullins et al. conducted a decision-analytic model utilizing data from two prospective, randomized, controlled trials. The estimated median daily treatment charges were $2888 for linezolid and $2993 for vancomycin. The incremental cost-effectiveness ratio for linezolid per life saved was $3600. The authors concluded that in the US, the higher acquisition cost of linezolid was largely offset by improved survival and reduced healthcare costs associated with improved survival [19]. In Brazil, a decision tree model was utilized to estimate the total cost per cured patient. The findings indicated that the cost per cured patient was approximately $3,109.50 for name-brand vancomycin, $2,642.90 for generic vancomycin, and $1,824.90 for linezolid. These results led to the conclusion that despite its higher unit price, linezolid demonstrated greater cost-effectiveness compared to vancomycin [20]. A previous study conducted in Colombia, based on data from a singular randomized clinical trial, showcased that the total costs associated with a patient's cure were lower for LNZ compared to VMC. The cost-effectiveness ratio of linezolid relative to vancomycin was determined to be approximately US$1332 [11]. In this sense the strength of this study lies in the fact that it was based primarily on in the results of metanalysis of seven randomized clinical trials which constitutes the most recent evidence and with the highest level of evidence available regarding the effectiveness of LNZ compared to VMC.

The concept of net benefit involves evaluating the discounted lifetime costs (in US$) and discounted lifetime quality-adjusted life years (QALYs) on a unified scale. This approach proves particularly valuable when comparing multiple strategies, as it allows analysts and decision-makers to assess the expected net value of each strategy in a single measure, avoiding the need to analyze numerous incremental cost-effectiveness ratios across different options. According to decision theory principles, the optimal strategy is the one that yields the highest expected net benefit. In our analysis, LNZ emerged as the strategy with the highest expected net benefit. To quantify the value of perfect information, we calculated the expected value of perfect information (EVPI) for healthcare decision-makers in Colombia, utilizing a threshold of USD $5,180 per QALY. The EVPI represents the monetary difference in health gain associated with the uncertainty in the parameters (current available information) and the health gain without uncertainty in the parameters (based on a model assuming perfect information) [14]. The estimated overall expected value of perfect information (EVPI) per affected individual is approximately USD $2.22. Considering a yearly population of 1000 patients with ventilator-associated pneumonia (VAP) in Colombia, the overall EVPI per year is approximately USD $225. Therefore, the overall EVPI per year is relatively low. Any research costing more than this amount would not be considered an efficient utilization of resources, as the anticipated return on investment from the research is expected to be no higher than USD $22,253. As expected, the payer strategy-specific burden (PSB), which represents the financial risks borne by the payer, is higher in the VMZ group compared to LNZ. The PSB is calculated as the difference between the expected net benefit of the most cost-effective option and the expected net benefit of the decision option [17]. The PSB indicates to the payer the risk of choosing an option that is not the most cost-effective option, for 1000 annual cases for VAP in Colombia it was US$1040 is VMZ is selected.

Our estimates are reliable, given that our model was robust to variations in utilities transition probabilities and costs. Indeed, we decided to use utilities reported in a systematic review in order to have broader values and in more diverse populations. Variations in the values of these utilities in the probabilistic sensitivity analysis did not significantly change the calculated ICER or incremental net monetary benefit. Partial EVPI enables identification that only the cost of LNZ, contribute particularly to decision uncertainty. And as was expected, more clinical trials can perhaps increase the reliability of this association.

Our study has some limitations. We used a relative risk extracted from the literature and not estimated directly from our population. However, the results of the probabilistic sensitivity analysis confirmed the robustness of the model’s results. The relative risk was subjected to a probabilistic sensitivity analysis, as detailed above and as recommended by the Consolidated Health Economic Evaluation Reporting Standards (CHEERS) Statement [8].

In conclusion, LNZ is a cost-effective strategy for patients, ≥ 18 years of age, with VAP in Colombia. Our study provides evidence that can be used by decision-makers to improve clinical practice guidelines.

### Supplementary Information

**Additional file 1.**

## Data Availability

The data that support the findings of this study are available from the corresponding author, upon reasonable request.

## Abbreviations

WTP: Willingness to pay
RR: Relative risk
CHEERS: Consolidated Health Economic Evaluation Reporting Standards
