# Supplementary material for: Cost-effectiveness of linezolid to ventilator-associated pneumonia in Colombia
Source: BMC Infect Dis. 2024 Jan 18;24:98. doi: 10.1186/s12879-023-08961-y (PMC10795396; doi:10.1186/s12879-023-08961-y)

**Figure 1.** Net monetary Benefit vs. Willingness-to-Pay


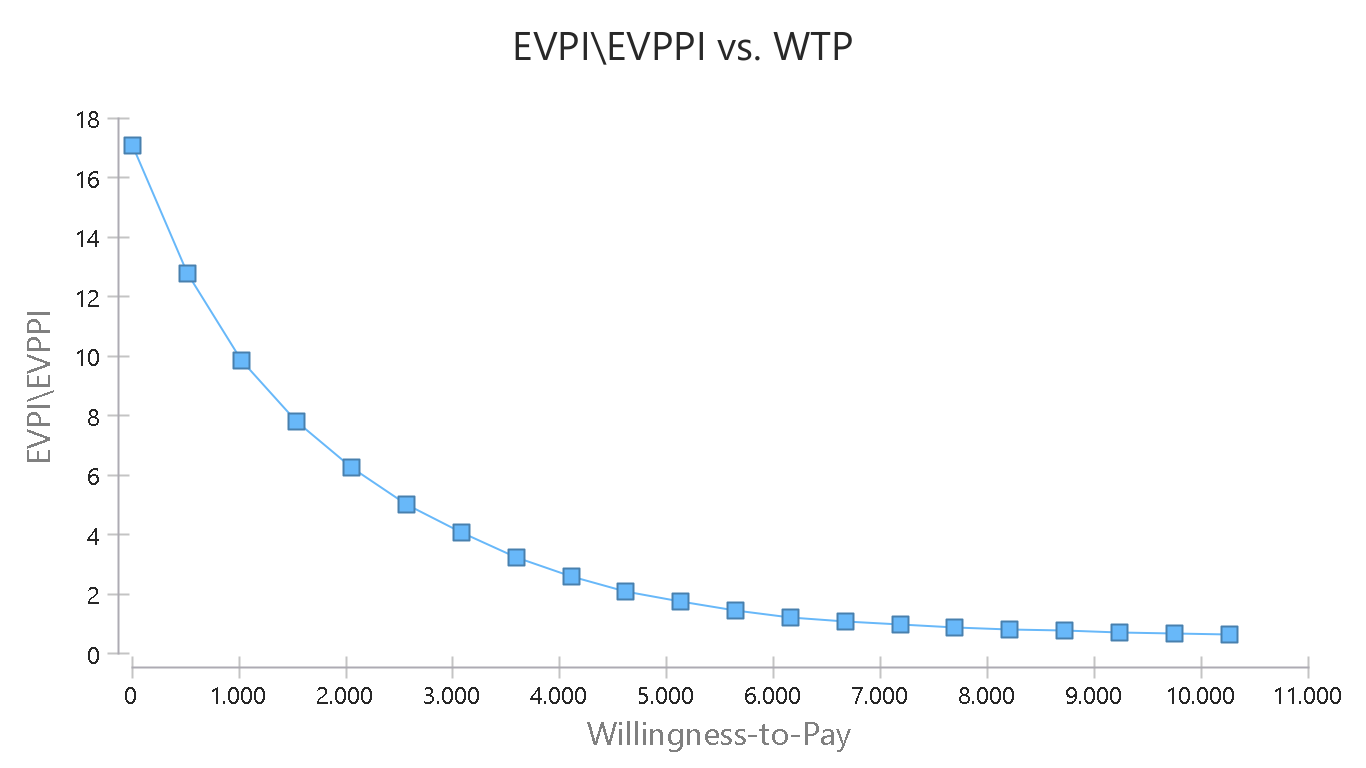


EVPI: expected value of perfect information

EVPPI: Expected Value of Partially Perfect Information

WTP: Willingness to pay

**Figure 2.** Distribution of incremental net monetary benefit


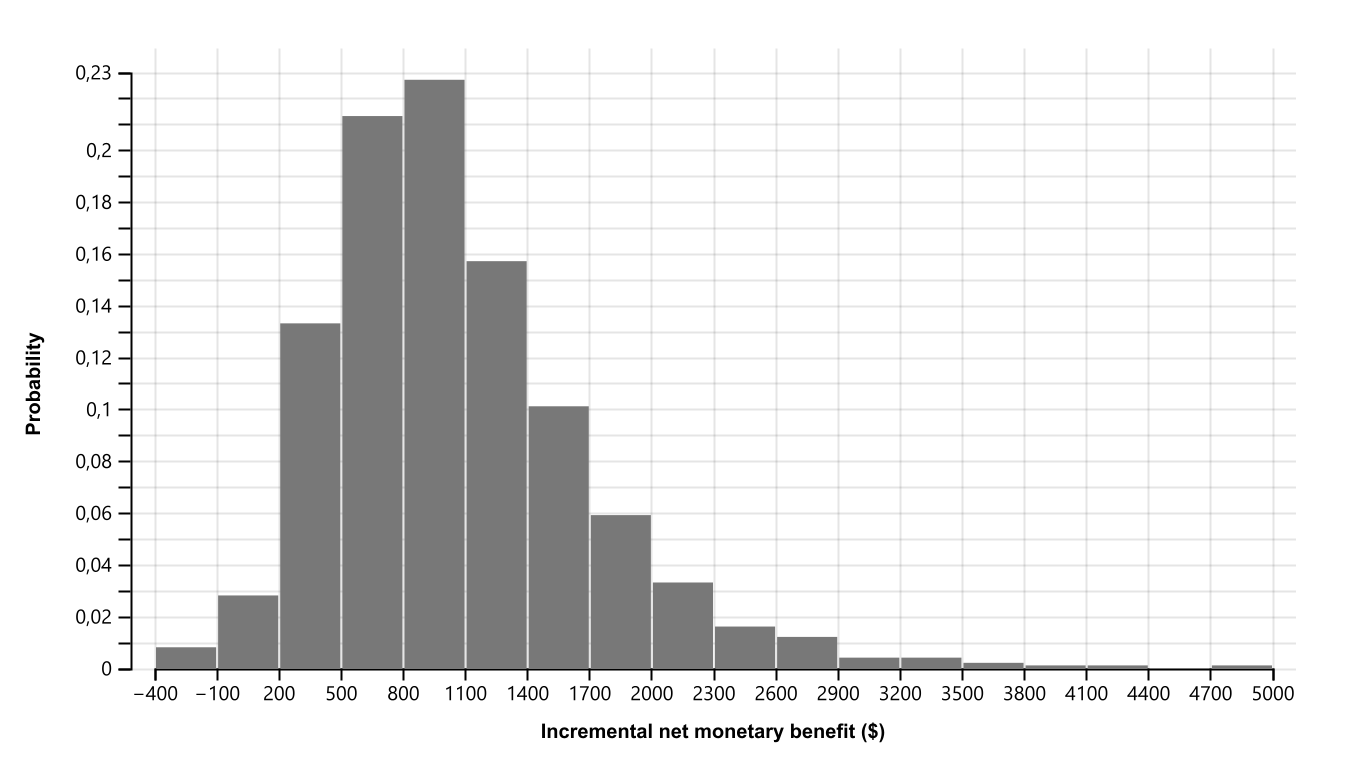

Supplement: Supplementary file 1 — Additional file 1. [file 12879_2023_8961_MOESM1_ESM.docx]
